# Quantifying the potential for bluetongue virus transmission in Danish cattle farms

**DOI:** 10.1038/s41598-019-49866-8

**Published:** 2019-09-17

**Authors:** Najmul Haider, Lene Jung Kjær, Henrik Skovgård, Søren Achim Nielsen, Rene Bødker

**Affiliations:** 1 National Veterinary Institute, Technical University of Denmark, Lyngby, Denmark; 2 Royal Veterinary College, University of London, London, United Kingdom; 3 Department of Veterinary and Animal Sciences, University of Copenhagen, Copenhagen, Denmark; 4 Department of Agroecology, University of Aarhus, Aarhus, Denmark; 5 Department of Science and Environment, University of Roskilde, Roskilde, Denmark

**Keywords:** Infectious diseases, Climate-change ecology, Entomology

## Abstract

We used a mechanistic transmission model to estimate the number of infectious bites (IBs) generated per bluetongue virus (BTV) infected host (cattle) using estimated hourly microclimatic temperatures at 22,004 Danish cattle farms for the period 2000–2016, and *Culicoides* midge abundance based on 1,453 light-trap collections during 2007–2016. We used a range of published estimates of the duration of the hosts’ infectious period and equations for the relationship between temperature and four key transmission parameters: extrinsic incubation period, daily vector survival rate, daily vector biting rate and host-to-vector transmission rate resulting in 147,456 combinations of daily IBs. More than 82% combinations of the parameter values predicted > 1 IBs per host. The mean IBs (10–90^th^ percentiles) for BTV per infectious host were 59 (0–73) during the transmission period. We estimated a maximum of 14,954 IBs per infectious host at some farms, while a best-case scenario suggested transmission was never possible at some farms. The use of different equations for the vector survival rate and host-to-vector transmission rates resulted in large uncertainty in the predictions. If BTV is introduced in Denmark, local transmission is very likely to occur. Vectors infected as late as mid-September (early autumn) can successfully transmit BTV to a new host until mid-November (late autumn).

## Introduction

Bluetongue virus (BTV) causes bluetongue disease (BT) – an important infection in ruminants and notifiable to the World Organization of Animal Health^1,2^. Symptoms of BT include weight loss, reduced milk yield and can lead to abortions and ultimately death. BTV circulates in a natural transmission cycle between insect vectors (*Culicoides spp*) and hosts (cattle, sheep, goat)^3^. BT is responsible for international trade restrictions on animals and animal products^1,2^, and has caused global losses of an estimated 2.6 billion EUR a year^2^. Six different strains of BTV belonging to 8 serotypes (BTV-1, BTV-2, BTV-4, BTV-6, BTV-8, BTV-9, BTV-11, BTV-16) and a vaccine serotype (BTV-14) have been reported in Europe since 2006^4^, and these viruses in combination have caused the most severe outbreaks of BT ever reported, resulting in the death of over 1.5 million sheep^1,5^. In Denmark, BTV was first identified in 2007, and another 15 outbreaks were identified across the country in 2008^6^.

Estimating the vectorial capacity (VC) of a vector-borne disease (VBD) is a common approach used to evaluate the potential threat of a disease outbreak. The VC of BTV is defined as the number of new hosts infected from an infectious host being exposed to vectors for one day^7,8^. Values for VC can aid in estimating potential outbreak risks, the initial number of animals infected, and the time when local transmission could occur. VC can be used as a risk assessment tool, where higher VC values indicate a potentially faster transmission^3^. The VC can be summed over the entire infectious period (period of viremia) of the host to obtain the basic reproduction number of a disease, commonly known as R_0_^8^. This parameter is defined as the number of infectious bites (IB) resulting from the infectious host and thus potentially the number of infected individuals originating from the introduction of one infectious host into a naïve population until the host recovers or dies^1,3^. IBs are the sum of infectious bites originating from all the daily cohorts of biting midges attacking the host during its infectious period^7,8^. Daily IBs represent the maximum potential for transmission and the estimates produced may be used for targeted surveillance and import regulations duringrisk periods.

The VC of a VBD can be expressed as a function of the number of vectors per host (m), vector biting rate (a), daily vector survival rate (p), the extrinsic incubation period (EIP), and the time interval between ingestion of an infected blood meal and the vector’s ability to transmit the virus to a new host (n). A simple version of the VC, adapted from Garrett-Jones^9^ and originally developed from Ross-Macdonald’s model for Malaria^10^ is shown as:

$$VC=m\ast {a}^{2}\ast {p}^{n}\ast (\frac{1}{-\,\mathrm{ln}(p)})$$

where, VC = new infections disseminated per cattle per day, ma = the number of bites/host/day, a = the frequency of blood feeding on cattle (1 divided by the duration of the gonotrophic cycle in days), p = probability of daily survival, n = time from infection to infectivity in days in the vector and is usually estimated from the ambient temperature using a degree-day relationship. Thus, p^n^ = probability of a mosquito surviving to become infective, and the expected duration of life in days after becoming infectious = 1/−ln(p).

The duration of EIP, vector biting rate, and vector survival rate are all highly dependent on temperature^11,12^. The EIP has a very strong impact on the VC of a disease, as the VC is proportional to the survival rate raised to the power of EIP. Other parameters that influence the VC, which are not included in the VC equation, includes the transmission rate from host to vector and the transmission rate from vector to host (cattle). Recently, temperature-dependent equations for BTV transmission from host to vector were suggested for *C. imicola* and *C. bolitinos* (Meiswinkel)^13,14^.

The original and modified versions of the Ross-Macdonald-Jones equation are deterministic and assume a “fixed rate” for most of the biological parameters^15^, whereas simulation models use a range of values^14^. The typical EIP equation^7,16,17^ used for BTV modelling has two important aspects: i) there is a threshold temperature below which no development is possible^1,7,13,16,18^, ii) above this threshold, there is either a linear or a more complicated non-linear relationship between the temperature and virus development in the vector^19^. The minimum threshold temperatures for virus development are often derived from a series of experiments conducted at constant temperatures^7,16–18^, after which a model is fitted to the data to predict development rates at other temperatures. In reality the threshold temperature may be much lower^12,19^ and temperatures climbing beyond this threshold may result in quicker virus development, followed by a period of more steady development^12^. The widely used equations in R_0_ modelling^7,16,17^ are likely to be less reliable at very high and very low temperature ranges, especially around the lower and upper threshold temperatures^19^.

Equations for the daily survival rate of *Culicoides* have mostly been developed from natural capture-release studies^18,20^. Some of these equations differ substantially in their estimates of daily survival rates, for example Wittmann *et al*.^16^ and Gerry & Mullens^18^. Furthermore, some researchers assume the daily survival rate is independent of age^20^, while others suggest it depends on age^21^.

Arthropods are poikilothermic, thus the environmental temperature affects the rate at which an arbovirus is able to replicate to a transmissible level within an arthropod vector^12^. Female biting midges spend almost 90% of their lifetime resting while digesting blood meals and developing eggs and the remaining period is for searching for a host for blood meals and mating^7,22^. The temperature, to which insects are exposed during resting are therefore important when modelling VBDs. However, most models of *Culicoides*-borne diseases^1,5,7,11,12,23,24^ predict the VC or R_0_ using meteorological temperature rather than the actual temperature in the microclimatic environment where the vectors rest. Microclimatic habitats are in general warmer than the estimates recorded by standard meteorological institutions^25^. Studies in Scandinavian climates have shown that microclimatic temperatures are warmer during the day and cooler during the night compared to recordings from nearby meteorological weather stations, and that the differences significantly affect the rate of virus development in *Culicoides* as well as the duration of the transmission season^25,26^. Even if the daily average meteorological temperatures and the daily average microclimatic temperatures were the same, the presence of threshold minimum temperatures for virus development and a non-linear relationship with temperatures above that threshold mean that the decreased speed of virus development during the night cannot compensate for the increase during the day. This means that models relying on meteorological temperature may underestimate development rates. Furthermore, many of the models used in estimating VBD transmission are based on monthly or daily mean temperatures^7,12,23^. In reality, insects do not experience a “mean temperature”, but are instead exposed to changing temperatures varying day by day and throughout the day^27^. Therefore, hourly temperatures at the vector resting sites can better capture the impact on VBD when modelling temperature-sensitive parameters.

*Culicoides imicola* was previously considered the main vector for BTV transmission in southern Europe^5,28^. Recently, BTV was isolated from wild specimens of *Culicoides obsoletus* (Meigen), *C. scoticus* (Downes & Kettle) and specimens of the Pulicaris sp.^28–31^. Identifying *Culicoides* species based on their morphological characteristics is difficult. Therefore, the term ‘ensemble’ is here suggested to denote a group of sympatric species for which morphological identification is sometimes difficult or not possible without phylogenetic analysis^32^. Obsoletus ensemble refers here to both the Obsoletus group and *C. dewulfi*, and includes *C. obsoletus*, *C. scoticus*, *C. montanus* (Shakirzjanova), *C. chiopterus* (Meigen) and *C. dewulfi*. The Pulicaris ensemble includes *C. pulicaris* (Linnaeus) and *C. punctatus* (Meigen)^32,33^. In this manuscript, we used both Obsoletus and Pulicaris ensemble abundance data to estimate the IBs with BTV resulting from an infectious host.

Many studies ignore the role of *C. pulicaris* in BTV transmission^1,34^ despite the Pulicaris ensemble comprising some of the most abundant vectors in “*imicola*-free” BT affected regions of Europe. Recent studies propose species within this ensemble to be potential vectors of BTV^29,30,33,35,36^. Experimental studies on oral susceptibility to BTV showed similar rates of infection in both the Obsoletus and Pulicaris ensembles (7.4% vs 13%)^30^. Another study using field caught specimens identified higher prevalence of BTV in the samples of the Pulicaris ensembles compared to that of the Obsoletus ensembles (57% vs. 46%)^36^. However, a large German study on field caught specimens showed a significantly higher number of Obsoletus ensembles being positive for BTV than Pulicaris ensembles (0.034% vs. 0.0002%)^29^.

The objective of this study was to use a mechanistic transmission model to: i) estimate the potential number of IBs per infectious host resulting from an introduction of BTV at farms in Denmark at any given day by including known parameter estimates and equations together with national vector surveillance and meteorological data; ii) quantify the uncertainty associated with each parameter.

## Methods

### Estimating microclimatic temperatures in Denmark, 2000–2016

**Figure 1:**
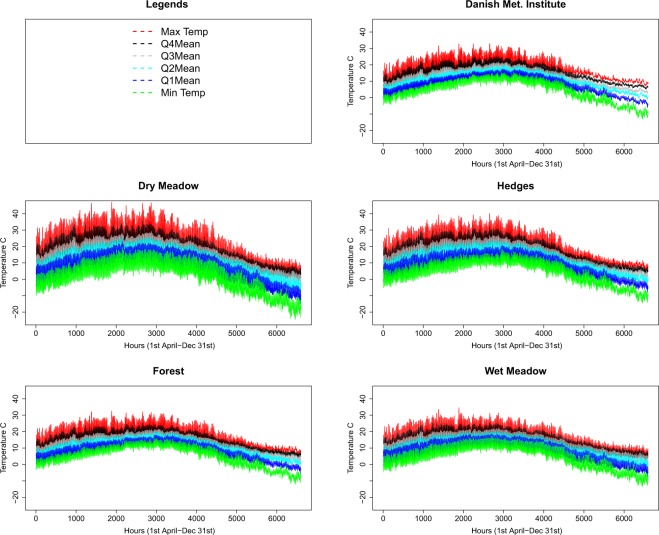
Six hourly microclimatic temperature series from April 1^st^ to December 31^st^ estimated at four potential insect habitats surrounding 22,004 Danish cattle farms and the temperature modelled by the Danish Meteorological Institute. Q4Mean, Q3Mean, Q2Mean and Q1Mean are the means of the fourth, third, second and first quantile observations.

We obtained hourly meteorological parameters (temperature, solar radiation, humidity, wind speed) for the period 2000–2016 from the Danish Meteorological Institute (DMI) at 320 grid points across Denmark. We considered the nearest grid point to each of the 22,004 cattle farms to be the temperature of that farm. The type of land cover within a 500 m radius of each cattle farm was quantified using CORINE Land Cover^37^. The land cover was reclassified as dry meadow (83%), hedges (6%), wet meadow (3%), and forest (3%)^26^. Using a previously published microclimatic model for Denmark^25,26^, these meteorological parameters were converted to estimates of hourly microclimatic temperatures for the period of April 1^st^ to December 31^st^ for each of the four different microclimatic habitats. This resulted in four different hourly temperatures for the 22,004 cattle farms over the 17 years. From each of these four microclimatic series we calculated the hourly mean of the first quantiles (>minimum and <25^th^ percentile), mean of the second quantiles (≥25^th^ percentile <50^th^ percentile), mean of the third quantiles (≥50^th^ percentile <75^th^ percentile) and mean of the fourth quantiles (≥75^th^ percentile) over the 17-year period. To quantify the impact of temperature on IBs, we ran our model with all four series of hourly temperatures for each of the four habitats in all combinations with the other model parameter settings (different equations of EIPs, daily survival rate, biting rate, host-to-vector and vector-to-host transmission rate). In addition, to quantify the worst- and best-case scenario, we also identified the maximum and minimum temperature of all farms each hour and for each microclimatic habitat, respectively from this 17-year hourly temperature dataset. An annual average of the six temperature series from each habitat data is presented in Fig. 1.

### Culicoides density data

Danish researchers have carried out a number of surveillance and research projects on abundance of biting midges across Denmark since 2007. Surveillance was conducted at 22 sites over the winter of 2007, 2008 and 2010 to identify the vector-free season. To monitor *Culicoides* in the warm season, 12 farm sites were randomly selected across Denmark during 2008 and 2009, and each site was sampled 29 times. In four of these 12 farms midges were collected for two nights every week during 2012, and three nights every week after this. From 2012 onwards, midges have been monitored in three of these four sites as a part of the national monitoring of *Culicoides*, and surveillance information is updated on the vector surveillance website: http://www.myggetal.dk/. Surveillance data were available for the period 2012–2016. In addition, collections were taken over one night at 251 farms in 2008 and 124 farms in 2009 from different regions of the country. The Onderstepoort light trap was used to collect the biting midges. Where collections were made on more than one night, we calculated the daily mean number assuming the trap had collected the same number of midges each day. Details of the counting and species-identification methods are described by Lassen *et al*.^38^, while surveillance collections are only identified to the two ensemble levels. In this study, we assumed the used Ondersteport trap acts like a host and would attract the same number of *Culicoides* as one host will attract as also assumed by Guis *et al*.^39^. Thus, we prepare a dataset with counts of biting midges for each week of different years.

**Figure 2:**
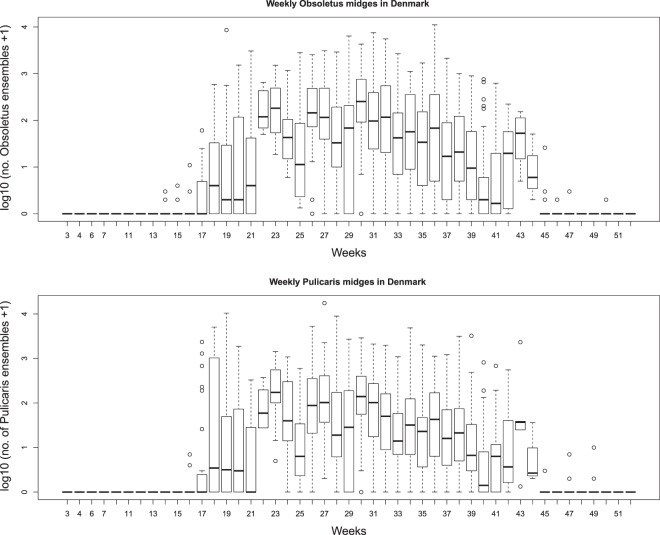
Boxplot of average weekly *Pulicaris* and *Obsoletus* ensemble abundance in Denmark based on collections during 2007–2016. The bottom and top of the box indicate the first and third quartiles *Culicoides* number; the band inside the box is the median *Culicoides* number. and the whiskers the lowest and highest data points still within 1.5 times the interquartile range of the respective lower and upper quartiles. The dots outside the box are outliers.

From the obtained *Culicoides* abundance data, we calculated the mean of the first quantiles (>minimum and <25^th^ percentile), mean of the second quantiles (≥25^th^ percentile <50^th^ percentile), mean of the third quantiles (≥50^th^ percentile <75^th^ percentile) and mean of the fourth quantiles (≥75^th^ percentile) of the number of Obsoletus and Pulicaris ensembles separately from the daily trap data. We then generated a daily abundance for each of the four estimates for each of the two species ensembles by assuming the abundance would be the same on each day of the week. Finally, we smoothed each of these eight daily *Culicoides* abundance series by a 15-day running average and used these daily vector abundances on cattle farms in Denmark as estimates of abundance for an average vector season. The resulting estimates of IBs are therefore averages for Denmark ignoring spatial variation as well as year-to-year variation in both microclimatic temperatures and vector abundance. In addition, to quantify the worst and best case scenario, we identified the maximum and minimum number of Obsoletus and Pulicaris ensembles each week from the 10-years *Culicoides* dataset and smoothed them with 15-day running averages. We present summaries of vector abundance data in Fig. 2.

### The equations used

The identified equations used in the transmission model and their references are listed in Table 1 and Fig. S1.The four different EIP equations are referred to as EIP equation I^7^, EIP equation II^16^, EIP equation III^16^, and EIP equation IV^12^. The four equations have a threshold temperature for start of virus replication, which is 10.4, 11.0, 14.1 and 13.3 °C respectively. We refer to the three different daily insect survival rate equations as Survival rate equation I^16^, Survival rate equation II^18^ and Survival rate equation III^18^. Survival rate equation I shows a very high survival rate (e.g. 90% at 30 °C) whereas survival rate equation III shows a low survival rate (e.g. 6% at 30 °C). All the survival rates are based on the assumption that midge daily survival rate is independent of age. We used an equation for frequency of blood feeding by the biting midges on the host^7^.

**Table 1:** Parameters used in modelling bluetongue virus transmission.

Parameters	Equations	Sources/References	Name in the manuscript
Survival rate of *Culicoides* biting midges	1 − (0.015*exp(0.063*Temp))	Wittmann *et al. (2002)*^16^	Survival rate equation I
	EXP (−1/(111.84*EXP(−0.1547*Tmean)))	Gerry & Mullens (2000)^18^	Survival rate equation II
	1 − (0.009* exp (0.16*Tmean))	Bessell *et al. (2016)*^49^ Gerry & Mullens (2000)^18^	Survival rate equation III
1/Extrinsic Incubation Period	((0.0003T(T − 10.4)) (BTV)	Mullens *et al*. (2004)^7^	EIP-I
	0.0069T −0.0636 (BTV 10)	Wittmann *et al. (2002)*^16^	EIP-II
	0.0113T − 0.1419 (BTV 16)	Wittmann *et al. (2002)*^16^	EIP-III
	0.019 *(T − 13.3) (BTV 9)	Wilson & Mellor (2009)^17^ Carpenter *et al*. (2011)^12^	EIP-IV
Biting rate (blood meal digestion)	0.0002T(T − 37) (41.9 − T)^1/2.7^	Mullens *et al*. (2004)^7^	Biting rate
Rate of transmission from host-to-vector	0.0003699 exp(0.1725T) *(originally developed for C. imicola)*	Paweska *et al. (2002)*^13^ Turner *et al*. (2013)^14^	Host-to-vector I
	0.005465 exp(0.159T) (*originally developed for C. bolitinos*)		Host-to-vector II
	Fixed rate (Used for Obsoletus ensemble only)	0.071 (Median of a range of transmission from host-to-vector used by Szmaragd *et al*. 2009, Gubbins *et al*. 2008 and)^1,23^	Host-to-vector III
Rate of transmission from vector-to-host	0.80	O’Connell (2002)^51^	Vector-to-host

Most BTV transmission models use a fixed rate of transmission from host to vector^1,14,23^. In this manuscript, we used two temperature dependent equations described by Turner *et al*.^14^ from the study of Paweska *et al*.^13^ and one fixed rate of transmission (median value from a range of distributions) from host to vector used by Gubbins *et al*.^1^ and Szmaragd *et al*.^23^ (Table 1). We used these three equations for both Obsoletus and Pulicaris ensembles considering that the rate of BTV transmission from host to vector is the same in both species. We refer to the rate of BTV transmission from host to vector as Host-vector transmission I (developed for *C. imicola)*^13,14^, Host-vector transmission II (developed for *C. bolitinos*)^13,14^ and Host-vector transmission III (fixed rate) (Table 1). We define the transmission season as the period when a vector ingesting an infected blood meal will survive to become infectious and infect a new host.

### Mechanistic model for estimating the number of daily IBs by *Culicoides* insects

We used a mechanistic model to estimate the potential number of IBs originating from one infectious host via *Culicoides*. This is a biological process-driven model based on the above-mentioned parameters. The model has previously been described^40^, and a similar model for another VBD, *Setaria tundra* (Filarioidea: Onchocercidae), has been described by Haider *et al*.^41^.

The model was designed to follow daily cohorts of biting midges throughout the season at hourly temperatures estimated for the four habitats: dry meadow, wet meadow, hedges and forest. In the model, biting midges took a blood meal infected with BTV, rested until the gonotrophic cycle was completed and then successfully took a new blood meal. Completion of the EIP was solely dependent on the hourly temperature experienced by the cohort of midges on each consequtive day. After the EIP was complete, we assumed that all subsequent bites by the biting midges were infectious until all vectors in the cohort were dead. We used a rate summation model in which the EIP or blood meal digestion rate was calculated hourly and summed up daily until the virus development/blood meal digestionwas complete.

The steps in the model are described below^41^.

1. The daily survival rates for *Culicoides* midges were calcuated using the daily mean temperaure recorded/predicted by the Danish Meteorological Institute and the equations listed in Table 1. We assumed a maximum survival time of 60 d for *Culicodes* biting midges in Denmark with a daily maximum survival rate of 90% and minimum survival rate of 1% (Table 1).
2. The model calculated the EIP for BTV (Table 1) based on successive hourly temperatures for each daily cohort and identified the date when the biting midges in each cohort became infectious, i.e. when the EIP was complete.
3. The model calculated and identified the dates when the vectors completed each gonotrophic cycle (Table 1) based on the successive hourly temperatures for each daily cohort. It was assumed that the biting midges would take a new blood meal immediately after the gonotrophic cycle was complete.
4. The model identified the date of the IBs in each cohort as the date when vectors bite after the EIP was complete. This date was then merged with information on the survival rate of biting midges to calculate how many vectors of the original cohort have survived until that day.
5. The model estimated the proportion of vectors that became infected while taking a blood meal. This was done seperately for the Obsoletus and Pulicaris ensembles by using two temperature-dependent formulae as well as one fixed rate (Table 1). This proportion was multiplied by the number of surviving vectors to estimate the total number of IBs produced by the cohorts.
6. The model assumed 80% of IBs would successfully infect the host by multiplying the total number of IBs by a factor of 0.80 (the rate of BTV transmission from vector to host, Table 1).
7. The model estimated the IBs for the Obsoletus and Pulicaris ensembles seperately, then summed them up to estimate the total number of IBs for each day.
8. We summed up the total number of IBs estimated per infectious host by summing all the IBs over the infectious period (20.6 d) and named it “daily IBs per infectious host” or simply “IBs per host”
9. For example, daily IBs of 10 per infectious host on August 1^st^ meant that if a newly infectious cow was introduced on a farm on August 1^st^, this would result in 10 new infectious bites originating from that cow. Hence, “daily” refers to the first day in the viremia period of the host, though infectious bites may originate from anytime during the host infectious period to vectors. Considering a duration of the infectious period of 20.6 d and a vector life span of 60 d, the last date that a new host could be infected from a bite from a vector infected by that infectious cow would be October 21^st^.

### Summary of infectious bite estimates

From the literature we identified four EIP equations for BTV, three equations for *Culicoides* daily survival rate, three equations for transmission from host to vector (two are temperature dependent and one is a fixed rate), one equation for biting rate and one rate for transmission of BTV from vector to host. We used these in the model by assuming that all equations were equally likely to capture the true relationship between temperature and the relevant outcome. We then ran the model in all possible combinations of the equations (4 × 3 × 3 × 1 × 1 = 36). Each of the 36 model combinations was then ran hourly with four distributions of microclimatic temperatures (mean of fourth, third, second, and first quantiles) for each of four potential insect resting habitats (dry meadow, hedges, forest and wet meadow), and four series of Culicoides abundance data (mean of fourth, third, second and first quantiles) in both vector ensemples (the Obsoletus and Pulicaris ensembles); in total (36 × 4 × 4 × 4 × 2 = ) 4608 daily combinations.

Danish cattle farms are surrounded by different habitats including 83% dry meadow, 6% hedges, 3% forest and 3% wet meadow^25^ on average. We considered that biting midges would rest randomly in the different habitats surrounding the farm (the larger proportion a specific habitat constitutes around a farm, the larger the proportion of vectors will be resting in that habitat). To adjust for different areas covered by each habitat we multiplied our model output by the IBs estimates (28 times, as there were approx. 28 times more dry meadow than forest or wet meadow) by those estimated from dry meadow temperature. Hedges were two times more abundant than forest or wet meadow and we multiplied the IBs 2 times with those estimated by hedges, and used the complete dataset including the IBs estimated with wet meadow and forest temperature in summary analysis. Finally, our daily estimated IBs resulted in 147,456 IBs.

To identify the worst- and best-case scenarios, we also ran the model with the maximum hourly temperatures and the daily maximum abundance of biting midges and the minimum hourly temperatures and the daily minimum abundance of biting midges, respectively. This gave another 144 daily estimates (4 EIP × 3 Survival rate × 3 rate of transmission from host to vector × 4 habitats) for each of the worst and best-case scenarios for both the Obsoletus ensample and the Pulicaris ensemble. We then summed the IBs estimated for the Obsoletus and Pulicaris ensembles to get the total IB from Culicoides vectors per day. We identified the worst-case scenario as the maximum value of IBs for each day from 144 different estimates. Likewise, we identified the best-case scenario as the daily minimum value of IBs for each day from 144 different estimates.

### Data analysis

We calculated summary statistics to report the daily mean temperature and 10–90^th^ percentiles at each of the four microclimatic habitats as well as the DMI weather stations. We also did this for the number of biting midges per day. We calculated the mean IBs and the 10–90^th^ percentiles from the 147,456 daily IBs. To quantify the impact of the variation in each of the three different input parameters (temperature, vector abundance, habitats etc.), we calculated the mean IBs for each category of that particular parameter while allowing all other parameters to vary in all remaining combinations. To quantify the uncertainty in each of the parameter equations (EIPs, survival rate, host to vector transmission rate), we ran the entire model for each equation one at the time and present the mean IBs for each equation. The mechanistic model for estimating the IBs per host was developed in SAS version 9.4^42^ and all summary analyses and plots were performed in R version 3.4.0^43^.

## Results

### Temperatures in Denmark

Figure 1 summarizes the microclimatic temperature data and data from DMI. The dry meadow was the warmest microhabitat with a mean (10–90^th^ percentile) summer (July and August) temperature of 17.9 °C (12.4–24.6 °C) compared to hedges 17.8 °C (13.9–23.1 °C), wet meadow 15.8 °C (13.3–19.4 °C) and forest 16.7 °C (14–19.4 °C). The mean (10–90^th^ percentiles) summer DMI temperature was 17.0 °C (14.6–19.6 °C).

### Culicoides vectors in Denmark

We identified 1,463 trap collections over 1–3 nights from 351 cattle farms across Denmark between 2007 and 2016. The mean number (and the10–90^th^ percentiles) of the Obsoletus ensemble was 204 (0–488) per night, and the mean number of the Pulicaris ensemble was 142 (0–288) per night. The number of vectors in the Obsoletus ensemble started to increase in early May, reaching a peak in July with a mean (10–90^th^ percentiles) of 388 (0–1,140) midges per night. The number of vectors in the Pulicaris ensemble started to increase in April and peaked in June with a mean (10–90^th^ percentiles) of 281 (1–578) midges per night (Fig. 2). We found four or five generations of Obsoletus using the annual average, with the first peak seen in May, the second in June, third in July, fourth in August and the fifth and final peak was surprisingly seen between late October and the beginning of November. Although not as clear as for the Obsoletus ensemble, five or six generations of Pulicaris ensembles were observed between April and November (Fig. S2).

### IBs

**Figure 3:**
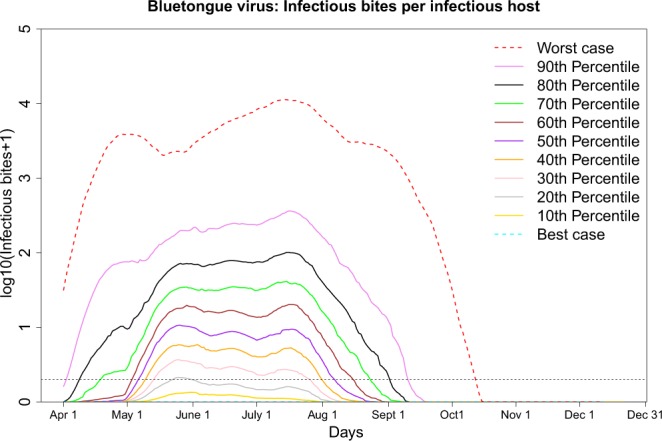
The number of infectious bites (IBs) with bluetongue virus per infectious host generated from 147,456 different combinations estimated by combining all the natural variation in temperature, vector abundance and resting habitats types as well as the uncertainty associated with equations used for extrinsic incubation period, daily survival rates, host-to-vector transmission rates, biting rates and vector-to-host transmission rates. Four different microclimatic temperatures were chosen from 2000–2016, as well as weekly vector density data from 1,453 trap collections across Denmark between 2007 and 2016. The worst-case scenario estimated the maximum of 14,954 IBs, whereas the best-case scenario estimated no transmission being possible in Denmark. The dotted horizontal line indicates IBs = 1 each day (log10 (1 + 1)). More than 82% combinations predicted >1 IBs per host at least one day of the transmission season.

Figure 3 summarizes the estimated number of IBs per host with BTV. The mean (10–90^th^ percentiles) number of IBs per host estimated for BTV in Denmark was 59 (0–73) per infectious host for the period April 1^st^ to October 31^st^, taking into consideration all possible combinations of parameter values. In the worst-case scenario, the daily maximum number of IBs per host was 14,954 IB, per infectious host, while no transmission was detected in the best-case scenario. More than 82% of the estimated values showed more than one IBs per host.

### Seasonality

While considering the median IBs of all combinations, the period when vectors could become infected and subsequently successfully transmit BTV lasted almost 4 months, starting in the first week of May and ending in the third week of August (Fig. 3). According to the median value of all combinations (50^th^ percentiles), the earliest possible day a cohort of *Culicoides* could become infected and successfully transmit BTV was in the first week of May with the resulting IBs being delivered up to 80.6 d later and the last day was in the third week of September (or second week of October based on the worst-case scenario; Fig. 3**)**.

We observed three peaks of IBs per host: at the end of May, middle of June, and the final and largest peak was observed between the second and fourth week of July. These peaks of transmission from host to vector correlated with the number of Obsoletus ensemble during the same period. The mean number of IBs with BTV per infectious host was 27 in April, 67 in May, 125 in June, 160 in July, 34 in August, and 3 in September (Fig. S2). We did not find a large discrepancy in the start or end of the season as estimated by the different equations (EIP, survival rate, host-to-vector transmission). However, different temperatures and vector abundances resulted in different durations of the transmission season. While the worst-case scenario showed almost 6 months of transmission, first quantile mean temperature showed around 3 months of transmission (mid-May to mid-August) and first quantile mean Obsoletus abundance showed less than 3 months of transmission (mid-May to the end of August).

### Uncertainty of parameter estimates in BTV transmission

**Figure 4:**
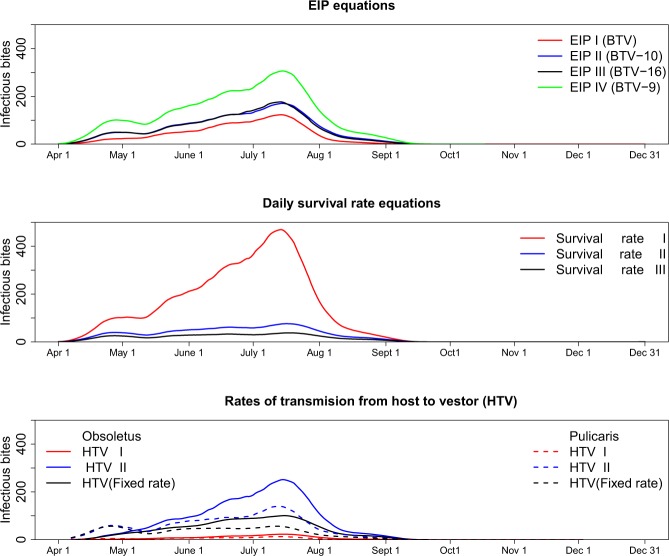
The mean infectious bites (IBs) per host estimated by different equations of three parameters including extrinsic incubation period (EIP), daily survival rates and rates of transmission from host-to-vector. The mean IBs for each equation of that particular parameter were estimated allowing all other parameters to vary in all remaining combinations. A large uncertainty is seen among three different equations of daily survival rate and among three rate of transmission from host-to-vector.

The mean IBs per infectious host during the transmission season (April to October) was estimated as 26 using EIP equation I, 42 using EIP equation II, 41 using EIP equation III, and 77 using EIP equation IV (Fig. 4). Survival rate equation I estimated much higher IBs (mean: 103) compared to survival rate equation II (mean: 23) and survival rate equation III (mean: 13) (Fig. 4).

The mean number of IBs by host-to-vector transmission estimated with equation II was more than ten-fold higher than with equation I (53.5 vs. 4.7 for Obsoletus ensembles and 33.9 vs. 3.1 for Pulicaris ensembles). The fixed rate host-to-vector transmission estimated a mean daily number of IBs of 28.1 for the Obsoletus ensemble and 19.7 for the Pulicaris ensemble (Fig. 4).

### Difference in BTV transmission potential due to natural variation in observed temperature, vector abundance and resting habitats

**Figure 5:**
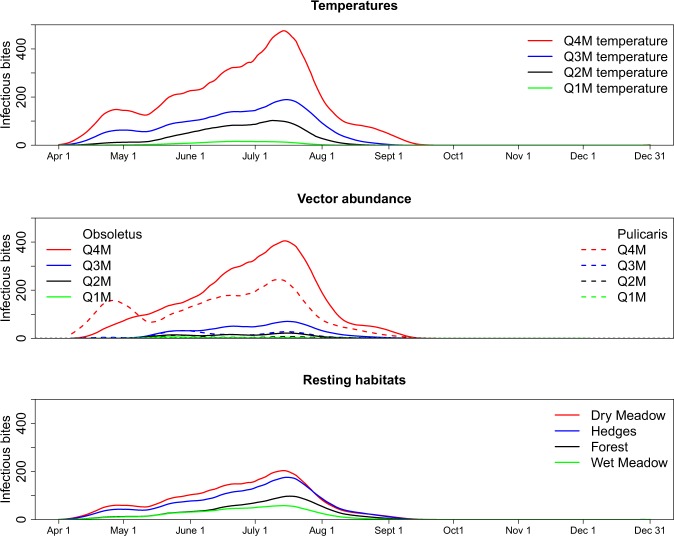
The mean infectious bites (IBs) per host estimated by different quantiles of observed temperatures, vector abundance and temperatures of resting habitats. The mean IBs for each quantiles of each particular parameter were estimated allowing all other parameters to vary in all remaining combinations. A large uncertainty is shown among four different quantiles of vector abundance. The *Pulicaris* ensemble appears early in the season and thus had higher estimates of IBs in spring. Q4M indicates mean of the fourth quantile (≥75^th^ percentile) observations, Q3M indicates mean of the third quantiles (≥50^th^ percentile <75^th^ percentile) observation and so on.

The number of IBs estimated from the fourth quantile mean temperature was three times higher than when estimated from the third quantile mean temperature, 52 times higher on average compared to estimates from the second quantile temperature, and 128 times higher compared to the first quantile temperature (Fig. 5).

In presence of favorable temperatures (when temperature is high enough to accomplish the EIP in 60 d), variation in vector abundance was the most influential parameter driving the IBs. The number of IBs estimated from the fourth quantile mean Obsoletus ensemble abundance was on average 13 times higher than the IBs estimated from the third quantile mean, 110 times higher than estimates from the second quantile, and 173 times higher than estimates from the first quantile (Fig. 5). For the abundance of the Pulicaris ensemble, the number of IBs estimated from the fourth quantile mean was on average 13 times higher than the IBs estimated from the third quantile mean, 346 times higher than estimates from the second quantile, and 653 times higher than estimates from the first quantile mean (Fig. 5).

The mean number of IBs per host was 49.1 when insects rested at dry meadow temperature, 40.7 at hedge temperature, 20.2 at forest temperature and 13.7 at wet meadow temperature (Fig. 5).

## Discussion

Our estimates of BTV IBs per host are based on the assumption that the identified equations used in BTV modelling are equally likely to be correct at estimating the number of IBs. Our model generated 147,456 different IBs by combining different parameter estimates of BTV in Denmark for each day of the potential transmission season (April 1^st^ to October 31^st^). Instead of calculating point estimates of IBs, we estimated a distribution of IBs per host for each day by combining all the natural variations (temperature, vector abundance and resting habitats types) and uncertainty associated with the different parameter equations (EIP, the daily survival rate, the host-to-vector transmission rate, biting rates and rate of transmission from vector- to- host). This allowed us to predict a wide range of IBs per host each day during the transmission season (April to October). Our estimates showed that only 18% of the combinations had less than one IBs per host during the entire season. The worst-case scenario predicted a very high average of 2,861 IBs for the entire season (April-October), with a maximum daily value of 14,954. The worst-case scenario was identified as the highest number of IBs estimated from the highest temperature recorded every hour over 17 years from any part of the country along with the highest number of biting midges recorded that week. The best-case scenario showed that no transmission would be possible, and was identified as the lowest number of IBs estimated with the lowest hourly temperatures over 17 years from any part of the country, leading to a prediction where virus development was never possible within the lifespan of the vector. However, both best-case and worst-case scenarios are applicable only to the farms with lowest and highest temperature each hour and vector abundance each week and should be considered only as extreme prediction scenarios for the country. The mean number of IBs per host for all combinations was 59 for the entire season, indicating that BTV has a high potential to spread if introduced^44^.

The number of IBs per host refers to the ability of the vector population to transmit a pathogen to the host population from an infectious host^7^ and is different from the basic reproduction rate or R_0_ in that the number of susceptible animals available is not accounted for. IBs are bites where infectious vira are transmitted to a host. However, a host may not be susceptible to BTV, or may already be infected or may have become immune. How IB translates into R_0_ on the given farm depends both on the availability of alternative hosts and on the proportion of vectors that disperse from the farm. The period of viremia in naturally infected animals varies for different strains of BTV with a range of 14–63 d and a mean of 20.6 d^44,45^. Our estimates therefore show that if BTV is introduced into the country, local spread is likely to occur. This finding is supported by empirical observations of BTV outbreaks in 2008 in Denmark, where a large number of animals were infected with BTV and local transmission was observed^6^.

We observed a large difference in estimates of BTV IBs per host due to natural variation in temperature, vector abundance and types of resting habitats. An increase of one quantile mean number of Obsoletus ensemble resulted in IBs that were at least 13 times higher than another quantiles. The 4^th^ quantiles mean Pulicaris ensemble estimated IBs that were 653 times higher than IBs estimated using the first quantile mean Pulicaris number. This is due to the large variation in *Culicoides* abundance observed between traps each week in different years or at different locations in Denmark. We found several generations of Obsoletus and Pulicaris ensembles during an average season. The peak of each vector generation coincided with the peak of IBs. In earlier studies, peaks of estimated R_0_ based on observed and predicted Obsoletus complex also coincided with a peak in the population of vectors^3^. The Pulicaris ensemble population peaks earlier in the spring, and there was a small peak in the daily IBs in the beginning of May due to vectors in the Pulicaris ensemble. Pulicaris ensemble has previously not been considered to be a competent vector in BTV transmission models^1,34^, although an experimental study found the Pulicaris ensemble to have similar vector competence as the Obsoletus ensemble^30^. The field collected specimens have showed higher prevalence of BTV in Obsoletus ensemble than in Pulicaris ensemble^29^. Pulicaris are abundant in the “non-imicola” region where BTV was detected^46^, and recent studies indicate that they play a role in BTV transmission^29,30^. In studies from southern and central Europe^3,31^, the Pulicaris ensemble populations only constitutes a small fraction of the biting midges populations, yet we found that in Denmark the Pulicaris ensemble populations were around half that of the total population of Obsoletus over the entire period. In general, Pulicaris are considered to be more abundant in Scandinavia than in central and southern Europe^47^ and if species in the Pulicaris ensemble are competent vectors, their high abundance makes it necessary to include them in transmission models for Denmark. Our model shows that Pulicaris ensemble could influence the infectious bites per host or R_0_ if the vectors are competent for BTV transmission. It is important to note that the host biting rates used to calculate IBs are based on the assumption that the number of female *Culicoides* collected in Onderstepoort light traps, reflects the biting rate, but light traps may both under- and overestimate actual biting rates^48^.

Temperature plays a critical role in driving many parameters including the EIP, survival rate, biting rate of vectors and host-to-vector transmission rate. In the best-case scenario, we used the minimum temperature among all the farms each hour to estimate the IBs of BTV which predicted transmission was not possible on any day. There was no transmission because virus development was not completed within the maximum lifespan of the vectors. In a situation like this, the number of insects becomes irrelevant; BTV transmission will not be possible despite high abundance of vectors. The model that used the first quantile mean temperature estimated a very low number of IBs per host because virus development was very slow, resulting in only few insects surviving to transmit the infections to new hosts when EIP was completed. One of the survival rates proposed by Gerry and Mullens (2000)^18,49^ (Survival rate III) estimated a very low number of IBs, because only a small proportion of vectors survived until the EIP was complete. We estimated higher numbers of IBs per host in dry meadow compared to other habitats, simply because the temperature on average was higher than in any other resting site.

In an earlier study from Denmark, Græsbøll *et al*. (2012) showed that the temperature and seasonality of vectors determined the period during which an incursion of BTV could lead to epidemic spread^45^. Furthermore, the authors concluded that within the transmission season, the number of affected animals will depend on the temperature and vector abundance, vector behavior and vector ability to locate hosts^45^. If the temperature remains favorable, the size of outbreaks will depend on the vector population. A generation of *Culicoides* will generally take 4–5 weeks to reach peak abundance. Our findings show that peaks of vector abundance coincide with peak IBs estimates, which indicate that seasonality of vectors drives the IBs.

During the 2008 BTV outbreak in Denmark, most of the cases were identified in late autumn (generally between September and October, while the last case was detected on November 17^th^)^6^. The date of some infections could possibly be earlier due to a delay in outbreak identification, but it has been unclear how BTV is transmitted at the low autumn temperatures found in Denmark. Our model used estimated microclimatic temperatures of potential vector habitats and showed that the last date when a cohort of vectors could be infected and be able to infect new hosts after completion of the EIP was in the second week of September (early autumn). Considering the maximum lifespan of *Culicoides* is 60 d, our model therefore showed that a successful transmission from vector to host is possible even in mid-November (late autumn) in Denmark.

We found a large variation in the estimated number of IB when using temperature-dependent equations for different parameters. We observed the largest variation in IBs per host between the equations used for the rate of BTV transmission from host to vector. The mean number of IBs estimated from the Host-vector transmission II equation was 10 fold higher IB than for equation I. The mean number of IBs estimated from survival rate equation I was 3.6 times higher than for equation II and 6.3 times higher than for survival rate equation III. The mean number of IBs estimated from EIP equation IV was 3.8 times higher than for EIP equation I, 1.9 times higher than for equation II and 1.8 times higher than for equation III. Such large uncertainty for a parameter will lead to substantial uncertainty in the outputs of a BTV R_0_ model, making it difficult to use model predictions to plan prevention and control strategies.

Although host to vector transmission is an important parameter, most models use a fixed number or a range of values for BTV transmission models. We used both temperatures dependent equations and a fixed rate for estimation of BTV transmission. The time it takes from when the virus enters the *Culicoides* to when it reaches a cell where it can replicate is unknown. However, virus attachment to and entry of a midgut cell within the *Culicoides* must occur before the peritrophic matrix (an acellular layer) form around the virus if a successful infection is to take place^50^. Therefore, we used the mean temperature of the day when the insect took the blood meal to calculate the temperature dependent probability of successful transmission of virus from host to vector. In total, we identified and used four equations for the EIP, three equations for the daily survival rate, two equations for the host to vector transmission rate and one equation for biting rates. However, there is a need to identify more precise parameters estimates for virus transmission in European *Culicoides*. Denmark experienced BTV outbreaks in two consecutive years in 2007 and 2008^6^. The IBs per host estimated from our model showed that only 18% of all estimates resulted in IBs per host less than 1. We suggest the quantitative predictions from modelling the transmission of BTV in northern Europe could be improved if more efforts were put into identifying and quantifying the correct relationships between temperature and *Culicoides* transmission parameters for BTV in European climates.

## Conclusion

We estimated 147,456 different IBs by combining different parameter estimates of BTV in Denmark for each day of the potential transmission season of which more than 82% of the estimated values showed more than one IBs per host. The mean (and 10–90^th^ percentiles) number of IBs was 59 (0–73) per infectious host over the transmission period. The best-case scenario in our model showed transmission was not possible. In the worst-case scenario, the transmission season lasted around 6 months (mid-April to mid-October), with a maximum number of IBs per host of 14,954. Therefore, it is likely that local spread will occur if BTV is introduced in Denmark. We identified a large uncertainty associated with the number of IBs estimated by different equations, including those for daily survival rate of *Culicoides* and host-to-vector transmission rates. We found temperature and the number of vectors to be the most influential factors in determining the BTV transmission (the daily number of IBs). Our model showed that the effective BTV transmission period is long in Denmark, and vectors infected as late as mid-September (early autumn) can successfully transmit BTV to a new host in mid-November (late autumn).

## Supplementary information

Supplementary info
