# Supplementary material for: Quantifying the potential for bluetongue virus transmission in Danish cattle farms
Source: Sci Rep. 2019 Sep 17;9:13466. doi: 10.1038/s41598-019-49866-8 (PMC6749064; doi:10.1038/s41598-019-49866-8)
Supplement: Supplementary file 1 — Supplementary info [file 41598_2019_49866_MOESM1_ESM.docx]

**Quantifying the potential for bluetongue virus transmission in Danish cattle farms**

# Najmul Haider^1,2*^, Lene Jung Kjær^1,3^, Henrik Skovgård^4^, Søren Achim Nielsen^5^, Rene Bødker^1,3^

^1^National Veterinary Institute, Technical University of Denmark

^2^Royal Veterinary College, University of London, United Kingdom

^3^Department of Veterinary and Animal Sciences, University of Copenhagen, Denmark

^4^Department of Agroecology, University of Aarhus, Denmark

^5^Department of Science and Environment, University of Roskilde, Denmark

*Corresponding author: [najha@vet.dtu.dk](mailto:najha@vet.dtu.dk)

**Additional/supplementary information:**

**Fig S1**: The relationship between temperature, pathogen development rate, daily survival rate, blood meal digestion rate and host-to-vector transmission rate in *Culicoides* used for the estimation of transmission potential*.* Maximum daily survival rate for vectors was set to 90% and minimum daily survival rate was set to 1%.

**Fig S2:** Boxplot of monthly number of infectious bites (IBs) per host infected with bluetongue virus (BTV), estimated for the date when a cohort of *Culicoides* could become infected and successfully transmit the BTV virus in Denmark. The highest numbers of IBs were recorded in July. The bottom and top of the box indicate the first and third quartiles value of all combination of IBs; the band inside the box is the median. The dots outside the box are outliers.
